# Supplementary material for: LRRK2 mediates haloperidol-induced changes in indirect pathway striatal projection neurons
Source: Mol Psychiatry. 2025 Apr 23;30(10):4473–86. doi: 10.1038/s41380-025-03030-z (PMC12436163; doi:10.1038/s41380-025-03030-z)
Supplement: Supplementary file 1 — Supplementary Figure 1 [file 41380_2025_3030_MOESM1_ESM.pdf]

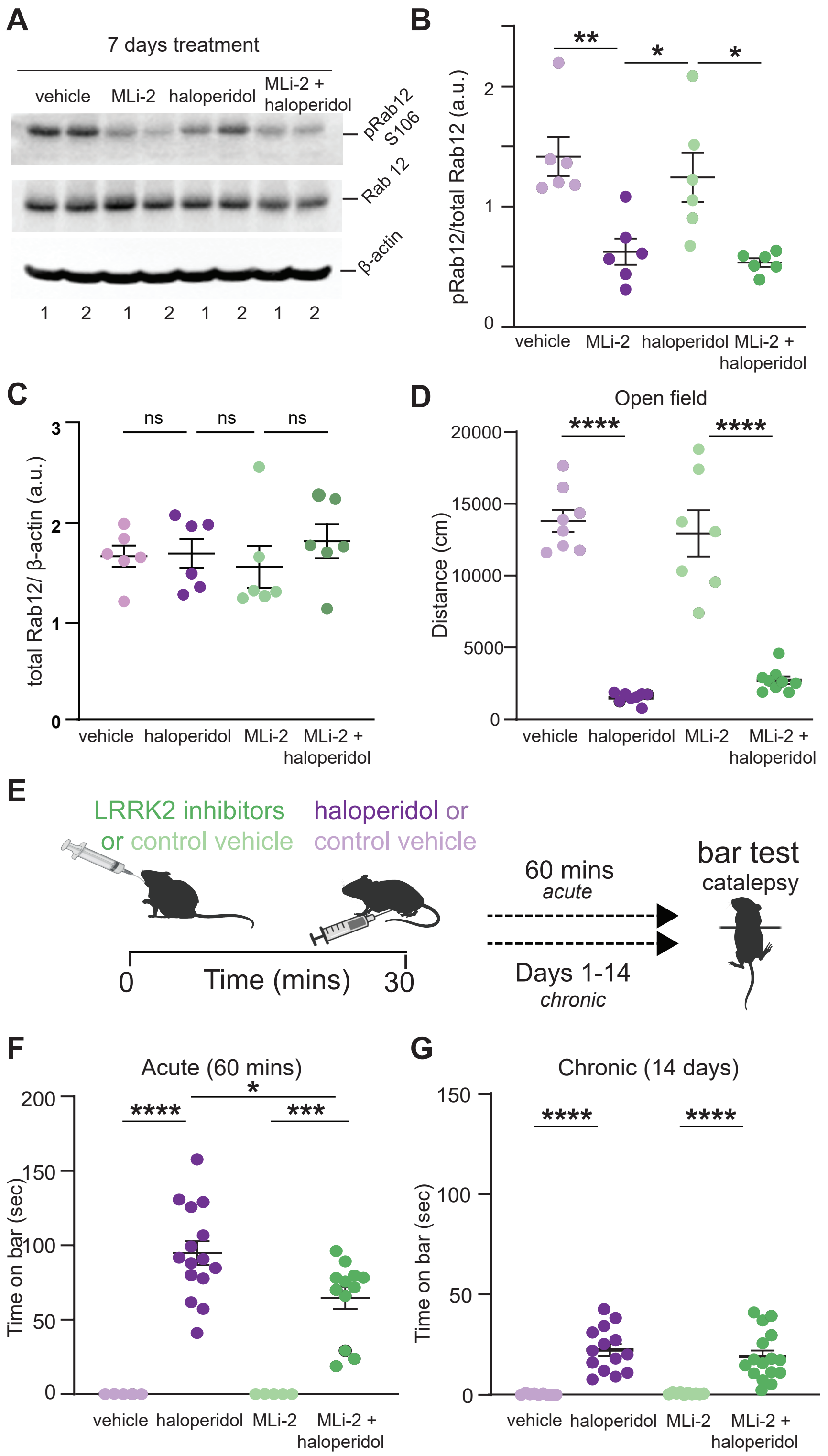

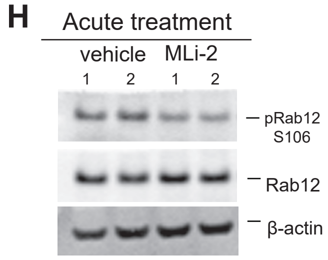

I

Treatment paradigm:	Acute (60 mins)	subchronic (7 days)	chronic (14 days)
Cataleptic response:	high	medium	low
LRRK2 inhibitor effect:	strong	strong	none

Supplementary Figure 1 (linked to Figure 1). LRRK2 mediates the effects of haloperidol on movement disruption

A. Western blot analysis of striatal extracts from mice across pharmacological treatments for 7 days, probed for pS106 Rab12 (LRRK2 kinase target), total Rab12, and β-actin.
B. Quantification of p-Rab12 band intensities normalized to total Rab12. n=6 mice
C. Quantification of total Rab12 band intensities normalized to total β-actin. n=6 mice
D. Distance traveled in the open field after 7 days of indicated pharmacological manipulations—N= 8, 8, 7, 9 mice, in order of groups presented.

E. Example of haloperidol and MLi-2 acute and chronic dosing schedule. Catalepsy was assessed 1 hour after the final haloperidol injection. Parts of the schematic were created with BioRender.com.
F. Catalepsy response of mice treated with haloperidol, MLI-2, or their combination. N=5, 15, 5, 12, in order of groups presented.
G. Cataleptic response after 14 days administration of haloperidol, MLi-2, or MLi-2 +haloperidol. N=9, 14, 10, 16.
H. Western blot analysis of striatal extracts from mice treated with MLI-2 or vehicle for 90 mins, as in F, probed for pS106 Rab12 (LRRK2 kinase target), total Rab12, and β-actin.
I. Table summarizing the magnitude of cataleptic response and the effects of LRRK2 inhibitor in cataleptic response across acute (1 hour), subchronic (7 days), and chronic (14 days) haloperidol treatment paradigms.

Data are represented as mean±SEM (error bars). Asterisks in B, C, D, F, and G denote statistical significance for Tukey's multiple comparison tests after one-way ANOVA. $*p<0.05$, $**p<0.01$, $****p < 0.0001$.
