# Supplementary material for: LRRK2 mediates haloperidol-induced changes in indirect pathway striatal projection neurons
Source: Mol Psychiatry. 2025 Apr 23;30(10):4473–86. doi: 10.1038/s41380-025-03030-z (PMC12436163; doi:10.1038/s41380-025-03030-z)
Supplement: Supplementary file 2 — Supplementary Figure 2 [file 41380_2025_3030_MOESM2_ESM.pdf]

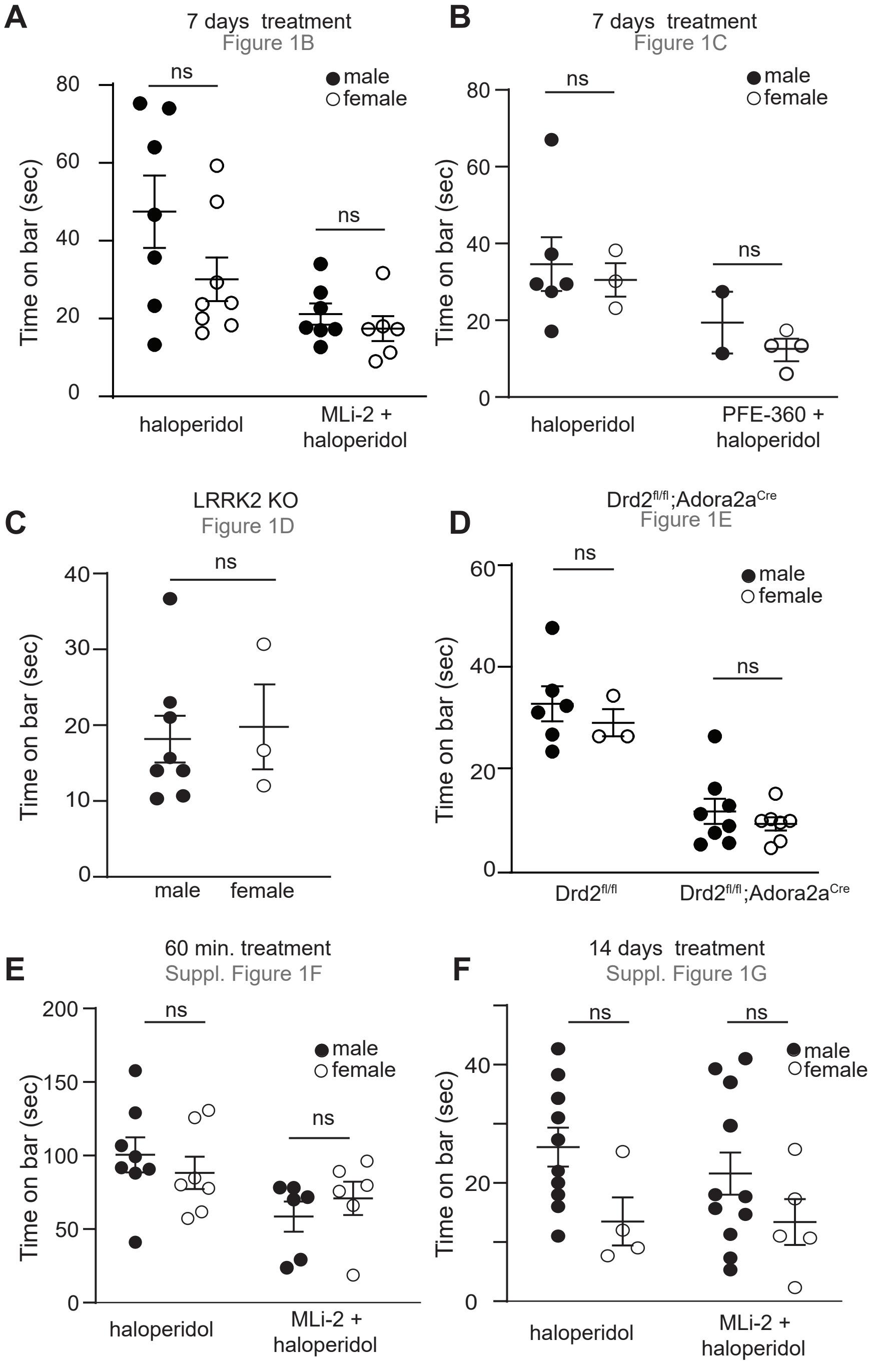
A
7 days treatment
Figure 1B
Time on bar (sec)
ns
ns
male
female
haloperidol
MLi-2 +
haloperidol
B
7 days treatment
Figure 1C
Time on bar (sec)
ns
ns
male
female
haloperidol
PFE-360 +
haloperidol
C
LRRK2 KO
Figure 1D
Time on bar (sec)
ns
male
female
D
Drd2fl/fl;Adora2aCre
Figure 1E
Time on bar (sec)
ns
ns
male
female
Drd2fl/fl
Drd2fl/fl;Adora2aCre
E
60 min. treatment
Suppl. Figure 1F
Time on bar (sec)
ns
ns
male
female
haloperidol
MLi-2 +
haloperidol
F
14 days treatment
Suppl. Figure 1G
Time on bar (sec)
ns
ns
male
female
haloperidol
MLi-2 +
haloperidol

Supplementary Figure 2 (linked to Figure 1). Haloperidol-induced cataleptic behaviors by sex

A-F. Summary graph showing the effect of sex across genotypes, pharmacological manipulations, and time points for data presented in Figure 1 and Supplementary Figure 1. Relevant figure panels are indicated. Data are represented as mean±SEM. ns, not significant for Tukey's multiple comparisons test except for C (unpaired t-test) after two-way ANOVA.
