# Supplementary material for: LRRK2 mediates haloperidol-induced changes in indirect pathway striatal projection neurons
Source: Mol Psychiatry. 2025 Apr 23;30(10):4473–86. doi: 10.1038/s41380-025-03030-z (PMC12436163; doi:10.1038/s41380-025-03030-z)
Supplement: Supplementary file 3 — Supplementary Figure 3 [file 41380_2025_3030_MOESM3_ESM.pdf]

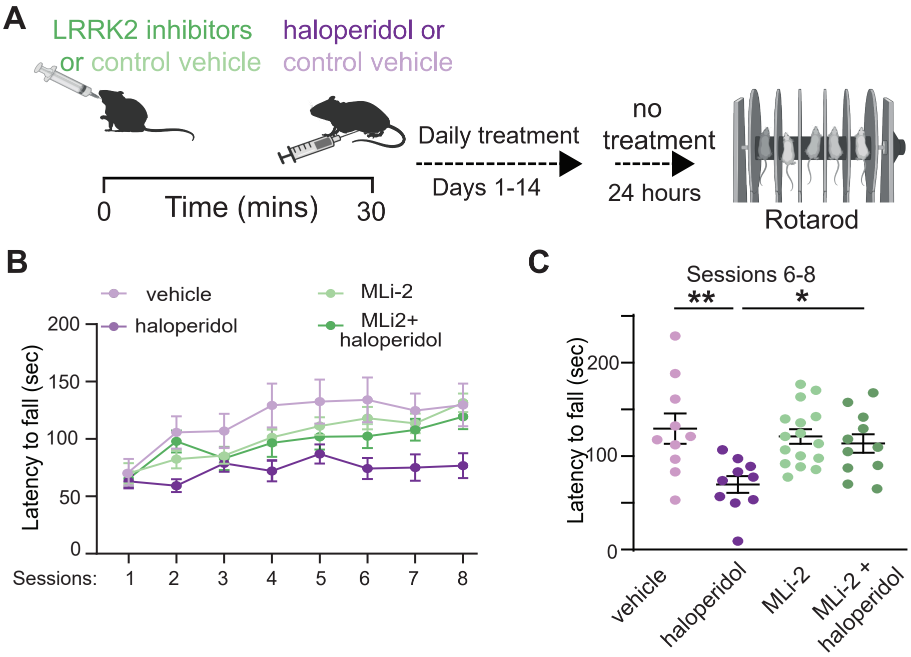

A
LRRK2 inhibitors
or control vehicle
haloperidol or
control vehicle
0
Time (mins)
30
Daily treatment
Days 1-14
no
treatment
24 hours
Rotarod
B
vehicle
haloperidol
MLi-2
MLi2+
haloperidol
Latency to fall (sec)
200
150
100
50
0
Sessions:
1
2
3
4
5
6
7
8
C
Sessions 6-8
**
*
Latency to fall (sec)
200
100
0
vehicle
haloperidol
MLi-2
MLi-2 +
haloperidol

Supplementary Figure 3 (linked to Figure 1). LRRK2 kinase inhibitors restore haloperidol-induced effects on striatal motor learning

A. Schematic of the experiment and treatment schedule; it contains schematics created with Biorender.com.
B. Accelerating rotarod performance (latency to fall) assessed over 8 daily sessions of 5 trials each. Mice received pharmacological compounds, as noted, and were evaluated 24 hours after the final haloperidol injection. n=10, 10, 16, 11, in the order are presented.
C. The average latency to fall in the last 3 sessions of B (Session 6-8). Asterisks show statistical significance for Tukey's multiple comparison tests after one-way ANOVA; $^{**}p < 0.01$, $^{*}p < 0.05$. data represent mean±SEM.
