# Supplementary material for: LRRK2 mediates haloperidol-induced changes in indirect pathway striatal projection neurons
Source: Mol Psychiatry. 2025 Apr 23;30(10):4473–86. doi: 10.1038/s41380-025-03030-z (PMC12436163; doi:10.1038/s41380-025-03030-z)
Supplement: Supplementary file 4 — Supplementary Figure 4 [file 41380_2025_3030_MOESM4_ESM.pdf]

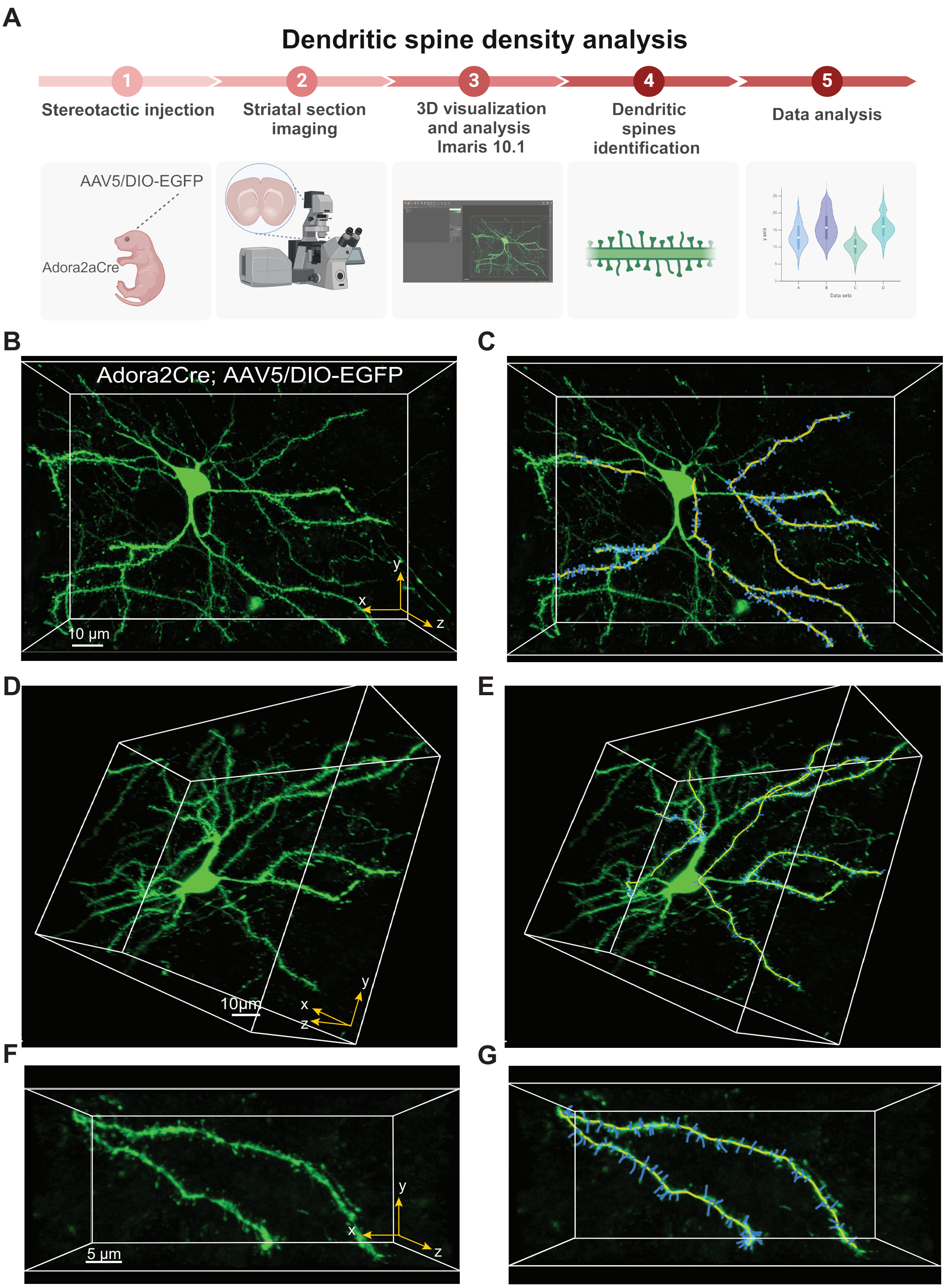
A
Dendritic spine density analysis
1
Stereotactic injection
2
Striatal section imaging
3
3D visualization and analysis Imaris 10.1
4
Dendritic spines identification
5
Data analysis
AAV5/DIO-EGFP
Adora2aCre
B
Adora2Cre; AAV5/DIO-EGFP
10 μm
x
y
z
C
D
10μm
E
F
5 μm
G

Supplementary Figure 4 (linked to Figure 2). Dendritic spine density measurement workflow

A. Workflow of experimental design for dendritic spine analysis using 3D visualization and analysis with Imaris 10.1. Created with BioRender.com.
B, C. Representative 3D volume rendering images of an *Adora2a*^{Cre} iSPN expressing AAV5/DIO-EGFP and the corresponding 3D Imaris filament tracer. Scale bar=10 µm
D, E. A different perspective angle for each x-y-z image in B, C. Scale bar=10 µm
F, G. Close-ups of dendritic segments from B, C. Scale bar=5 µm
