# Supplementary material for: LRRK2 mediates haloperidol-induced changes in indirect pathway striatal projection neurons
Source: Mol Psychiatry. 2025 Apr 23;30(10):4473–86. doi: 10.1038/s41380-025-03030-z (PMC12436163; doi:10.1038/s41380-025-03030-z)
Supplement: Supplementary file 5 — Supplementary Figure 5 [file 41380_2025_3030_MOESM5_ESM.pdf]

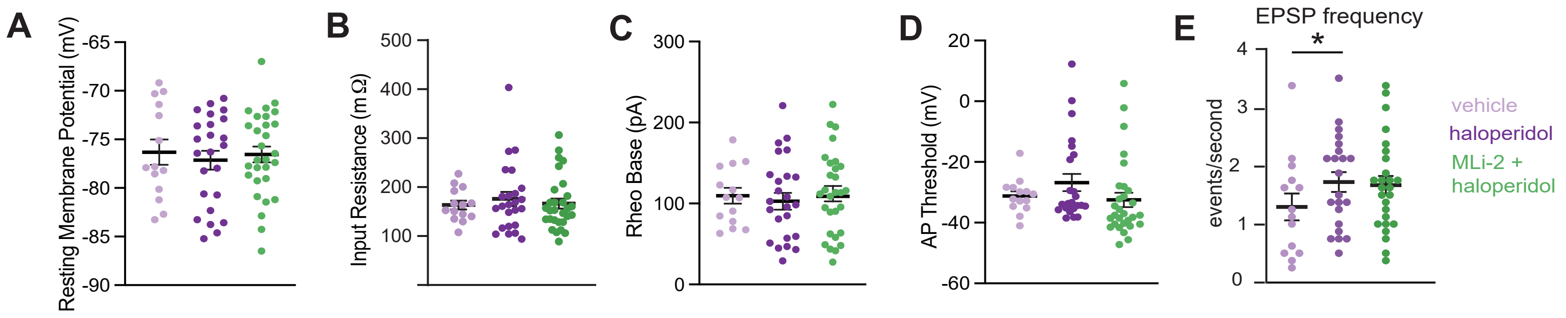
A
Resting Membrane Potential (mV)
-65
-70
-75
-80
-85
-90
B
Input Resistance (mΩ)
500
400
300
200
100
C
Rheo Base (pA)
300
200
100
0
D
AP Threshold (mV)
20
0
-20
-40
-60
E
EPSP frequency
*
events/second
4
3
2
1
0
vehicle
haloperidol
MLi-2 +
haloperidol

Supplementary Figure 5 (linked to Figure 2). Membrane properties and synaptic transmission of iSPNs.

A-D. General membrane properties for resting membrane potential, input resistance, rheobase, and action potential threshold. n=14-29 cells, 3-6 mice/condition. **E**. Average EPSP frequency across conditions. n=14-26 cells, 3-6 mice per condition. $p<0.05$, Mann Whitney U test. Data are represented as means ± SEM.
