# Supplementary material for: LRRK2 mediates haloperidol-induced changes in indirect pathway striatal projection neurons
Source: Mol Psychiatry. 2025 Apr 23;30(10):4473–86. doi: 10.1038/s41380-025-03030-z (PMC12436163; doi:10.1038/s41380-025-03030-z)
Supplement: Supplementary file 7 — Supplementary Figure 7 [file 41380_2025_3030_MOESM7_ESM.pdf]

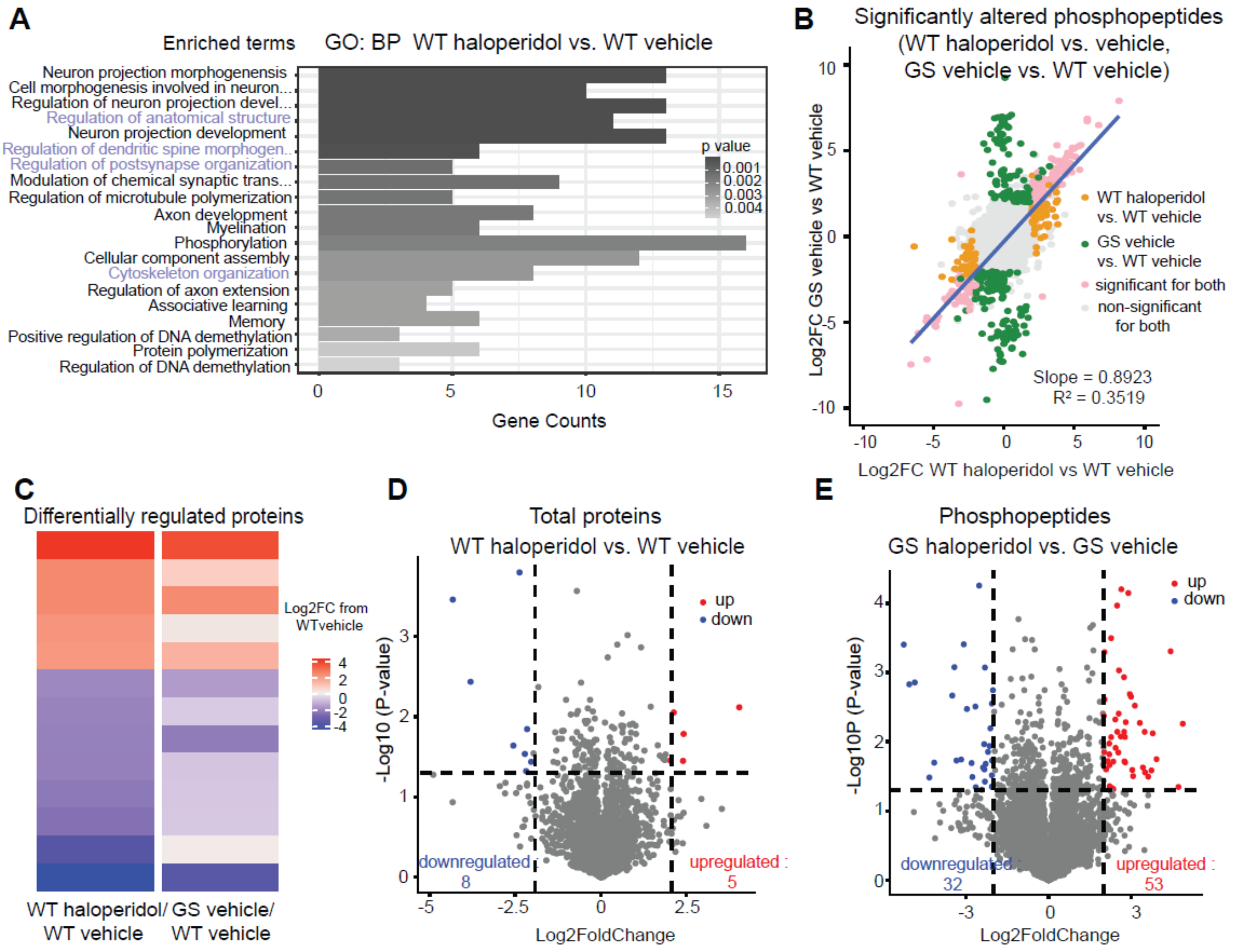

Supplementary Figure 7 (linked to Figure 3). Proteomic analysis of striatal extracts from haloperidol and vehicle-treated WT and GS mice

A. Results of GSEA for the Gene Ontology: Biological Processes gene set on genes with at least one significantly differentially regulated phosphopeptide for the WT haloperidol vs vehicle-treated comparison. All pathways displayed are significantly differently regulated (adjusted p-value ≤ 0.05 by Fisher's test). The length of bars shows the number of genes in the pathway whose phosphostate is differentially regulated, and bars are shaded by adjusted p-value. Highlighted pathways are related to known LRRK2 functions.
B. Correlation plot for Fig 4E, comparing GS vehicle/WT vehicle to WT haloperidol-vehicle effect size. All points mapped, all significant phosphopeptides (|Log2FC| > 2 and p-value ≤ 0.05 by multiple unpaired t-tests) for either comparison are highlighted and used for correlation. The blue line represents the line of best fit.
C. Heatmap of effect size (Log2FC) of either haloperidol treatment or LRRK2-GS for all proteins differentially expressed (|Log2FC| > 2 and p-value ≤ 0.05 multiple unpaired t-tests) compared to the vehicle-treated wild type condition in the wild type haloperidol- vs vehicle-treated comparison, mapped for both the wild type haloperidol- vs vehicle-treated comparison and the GS vs. wild type treated vehicle-treated comparison. Each bar represents a protein.
D. Volcano plot of the striatal proteome comparing the haloperidol- vs. vehicle-treated WT mice. Differentially regulated proteins (|Log2FC| > 2 and p-value ≤ 0.05 by multiple unpaired t-tests) are colored in red and blue for up- and downregulated, respectively.
E. Volcano plot of relative phosphopeptide for haloperidol- and vehicle-treated LRRK2-GS mutant mice. Phosphopeptides that are differentially regulated (|Log2FC| > 2 and p-value ≤ 0.05 by multiple unpaired t-tests) are colored red and blue for up and downregulated, respectively.
