# Supplementary material for: LRRK2 mediates haloperidol-induced changes in indirect pathway striatal projection neurons
Source: Mol Psychiatry. 2025 Apr 23;30(10):4473–86. doi: 10.1038/s41380-025-03030-z (PMC12436163; doi:10.1038/s41380-025-03030-z)
Supplement: Supplementary file 8 — Supplementary Figure 8 [file 41380_2025_3030_MOESM8_ESM.pdf]

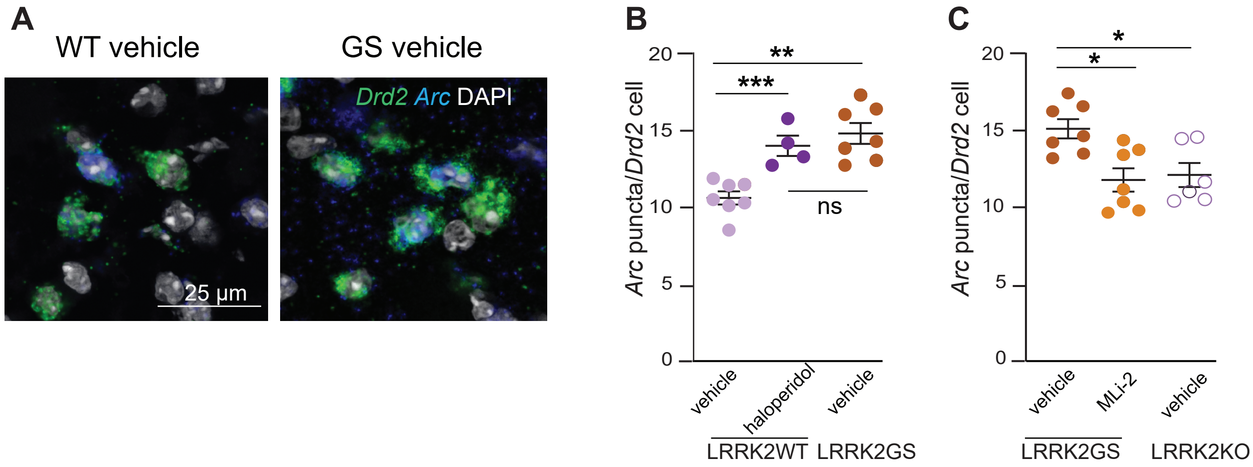

A
WT vehicle
GS vehicle
Drd2 Arc DAPI
25 µm
B
Arc puncta/Drd2 cell
20
15
10
5
0
***
**
ns
vehicle
haloperidol
vehicle
LRRK2WT
LRRK2GS
C
Arc puncta/Drd2 cell
20
15
10
5
0
*
*
vehicle
MLi-2
vehicle
LRRK2GS
LRRK2KO

Supplementary Figure 8 (linked to Figure 4). LRRK2 kinase activity underlies *Arc* increase in indirect pathway SPNs.

A. Example confocal images of *Arc* gene expression in iSPNs of LRRK2-WT and LRRK2-GS mice. Scale bar=25 μm
B. Quantification of the number of *Arc puncta* among *Drd2*-positive nuclei. LRRK2-WT mice were treated with haloperidol or vehicle,were treated with haloperidol or vehicle, and LRRK2-GS mice were treated with vehicle. Each dot represents the average number of *Arc* puncta among *Drd2*-positive nuclei from one striatal section, n=4-7 sections/3-4 mice/group.
C. Quantification of *Arc* puncta among *Drd2*-positive nuclei in LRRK2-GS mice treated with vehicle, MLi2, and LRRK2- KO mice treated with vehicle for 2 hours. N=6-7 sections/3-4 mice.

Data reflect mean±SEM. Asterisks in B and C reflect statistical significance for Tukey post-hoc comparisons after one-way ANOVA. *p < 0.05, **p< 0.01, ***p < 0.001.
