# Supplementary material for: LRRK2 mediates haloperidol-induced changes in indirect pathway striatal projection neurons
Source: Mol Psychiatry. 2025 Apr 23;30(10):4473–86. doi: 10.1038/s41380-025-03030-z (PMC12436163; doi:10.1038/s41380-025-03030-z)
Supplement: Supplementary file 9 — Supplementary Table 1 [file 41380_2025_3030_MOESM9_ESM.pdf]

RESOURCE TYPE	RESOURCE NAME	SOURCE	IDENTIFIER
Primary Antibodies	phospho-Rab12 (S106) 1:1000	Abcam	Cat# ab256487 RRID: AB_2884880
	Rab12 1:1000	Proteintech	Cat#18843-1-AP RRID: AB_10603469
	β-Actin 1:3000	Sigma-Aldrich	Cat#A5441 RRID: AB_476744
	anti-GFP 1:500	Invitrogen	Cat# A10262 RRID: AB_2534023
	anti-phospho-S6 Ribosomal protein (Ser236/236) 1:200	Cell signaling Technology	Cat#2211 RRID: AB_331679
	p-PKA 1:1000	Cell signaling Technology	Cat#9621 RRID: AB_331817
Secondary Antibodies	Goat anti-Rabbit IgG (H+L), HRP 1:2000	Thermo Fischer Scientific	Cat#656120 RRID: AB_2533967
	Goat anti-Mouse IgG (H+L), HRP 1:2000	Thermo Fischer Scientific	Cat#626520 RRID: AB_2533947
	Goat anti-Chicken IgY (H+L) Secondary Antibody, Alexa Fluor™ 488 1:300	Invitrogen	Cat# A11039 RRID: AB_2534096
	Donkey anti-Rabbit IgG (H+L) Highly Cross-Adsorbed Secondary Antibody, Alexa Fluor™ 647 1:300	Invitrogen	Cat#A31573 RRID: AB_2536183
Recombinant DNA Mouse models	AAV5-hSyn-DIO- EGFP ($7x10^{12}$vg/mL)	A gift from Bryan Roth (Addgene)	Cat#50457 RRID:Addgene_50457
	Tg (Drd2-EGFP)S118Gsat	MMRC	Cat#000230-UNC RRID:MMRRC_000230-UNC
	LRRK2 G2019S KI	Jackson Laboratory	Cat# 030961 RRID:IMSR_JAX:030961
	C57BL/6J	Jackson Laboratory	Cat# 000664 RRID:IMSR_JAX:000664
	LRRK2 KO mice	Jackson Laboratory	Cat# 016121 RRID:IMSR_JAX:016121
	Drd2fl/fl mice	Jackson Laboratory	Cat#020631 RRID:IMSR_JAX:020631
	Tg(Adora2a-cre)KG139Gsat	MMRRC Cat#031168- UCD	RRID:MMRRC_031168-UCD
Chemical compound, drug	(−)-Quinpirole hydrochloride	Millipore Sigma	Cat#Q102
	Haloperidol	Millipore Sigma	Cat#1303002
	MLi-2	Abcam,	Cat#ab254528
	Hydroxypropyl-β-Cyclodextran	Cayman	Cat#16169

PFE-360	MCE	Cat#Y-120085
hydroxypropyl cellulose	Millipore Sigma	Cat#191884
docusate sodium	Millipore Sigma	Cat#D1685
acetic acid	Millipore Sigma	Cat#A6283
Sodium Hydroxide 10N Concentrate	Fisher Scientific	Cat#SS267
Sodium chloride solution	Millipore Sigma	Cat#S5150
Phosphate buffered saline	Millipore Sigma	Cat#P5493
Halt Protease and Phosphatase Inhibitor Cocktail, EDTA-free (100X)	Thermo Scientific	Cat#78441
Tris Buffered Saline	Millipore Sigma	Cat#T5912
TWEEN® 20	Millipore Sigma	Cat#P1379
NuPAGE™ MES SDS Running Buffer (20X)	Invitrogen	Cat#NP0002
NuPAGE™ Antioxidant	Invitrogen	Cat#NP0005
NuPAGE™ LDS Sample Buffer (4X)	Invitrogen	Cat#NP0008
NuPAGE™ Sample Reducing Agent (10X)	Invitrogen	Cat#NP0009
Restore™ Western Blot Stripping Buffer	Thermo Scientific	Cat#21059
Cell Lysis Buffer (10X)	Cell Signaling,	Cat#9803
ProLong™ Diamond Antifade Mountant	Invitrogen	Cat#P36970
Sodium chloride	Millipore Sigma	Cat#S3014
Potassium chloride	Millipore Sigma	Cat#P9541
Sodium bicarbonate	Millipore Sigma	Cat#S5761
Sodium phosphate monobasic	Millipore Sigma	Cat#S3139
Calcium chloride	Millipore Sigma	Cat#C5670
D-(+)-Glucose	Millipore Sigma	Cat#G7021
Scopolamine hydrobromide	Tocris	Cat#1414

	Potassium gluconate	Millipore Sigma	Cat#1550001
	MgCl2	Millipore Sigma	Cat#M8266
	HEPES	Millipore Sigma	Cat#54457
Commercial assays or kit	Alexa Fluor 594 dye	Thermo Fisher Scientific	Cat#A10438
	Adenosine 5'- triphosphate magnesium salt	Millipore Sigma	Cat#A9187
	Guanosine 5'- triphosphate sodium salt hydrate	Millipore Sigma	Cat#51120
	Ethylene glycol-bis (2-aminoethylether)- N,N, N',N'-tetraacetic acid	Millipore Sigma	Cat#E3889
	Phosphocreatine disodium salt hydrate	Millipore Sigma	Cat#P7936
	RNAscope Multiplex Fluorescent Assay v2	ACD Bio	Cat# 323100
	RNA scope probe Mm- Arc-C3	ACD Bio	Cat#316911-C3
	RNA scope probe Mm-Nr4a1-C3	ACD Bio	Cat#423341-C3
	RNA scope probe Mm-Drd1a-C2	ACD Bio	Cat#406491-C2
	RNA scope probe Mm-Drd2	ACD Bio	Cat#406501
	RNA scope probe OPAL520	Akoya Biosciences	Cat#FP1487001KT
	Fluorophore Reagent OPAL570	Akoya Biosciences	Cat#FP1488001KT
	Fluorophore Reagent OPAL650	Akoya Biosciences	Cat#FP1496001KT
	NuPAGE™ Bis-Tris Mini Protein Gels, 4– 12%	Invitrogen NP0336BOX	Cat#NP0336BOX
	iBlot™ 2 Transfer Stacks, nitrocellulose	Invitrogen	Cat#IB23001
	Pierce™ BCA Protein Assay Kits	Thermo Scientific	Cat#23225
	NuPAGE™ Transfer Buffer	Invitrogen	Cat#NP0006
	Immobilon ECL Ultra Western HRP Substrate	Millipore	Cat#WBULS0500
	BLUeye Prestained Protein Ladder	Millipore Sigma	Cat#94964
	ProLong™ Diamond Antifade Mountant	Invitrogen	Cat#P36965
	OCT embedding medium	Fisher Scientific	Cat#4585

	Superfrost microscope slides	Fisher scientific	Cat#12-550-016
	DAPI Nucleic Acid Staining	Thermo Fischer Scientific	Cat#D1306
Software/ codes	Imaris 10.1	Bitplane Inc	RRID:SCR_007370
	GraphPad Prism 10	GraphPad Software Inc	RRID:SCR_002798
	Biorender	Biorender	RRID:SCR_018361
	ScanImage	MathWorks, Natick, MA	RRID:SCR_014307
	Enrichr	Ma'ayan Lab	RRID:SCR_001575
	Cell Profiler	Broad Institute	RRID:SCR_007358
	Image J	doi:10.1038/nmet h.2089	RRID:SCR_003070
	Limelight	Actimetrics	RRID:SCR_014254
	RStudio	RStudio, PBC, Boston, MA	RRID:SCR_000432
Data Set/ Protocol	MATLAB custom code	The MathWorks, Inc.	https://github.com/KozorovitskiyLaboratory/
	Primary data	Zenodo	https://doi.org/10.5281/zenodo.15092155
	Proteomics/ phosphoproteomics data	PRIDE	PXD053892
	Western blot images	Zenodo	https://doi.org/10.5281/zenodo.15092155
	IF images	BioStudies	S-BIAD1740
	smFISH images	BioStudies	S-BIAD1747.
	Genotyping protocol for mice	Protocols.io	dx.doi.org/10.17504/protocols.io.k xygx3mjwg8j/v1
	Catalepsy test (Bar test)	Protocols.io	dx.doi.org/10.17504/protocols.io.3 6wgq3ryolk5/v1
	Open field test	Protocols.io	dx.doi.org/10.17504/protocols.io.x 54v923m4l3e/v1
	Rotarod test	Protocols.io	dx.doi.org/10.17504/protocols.io.e q2lywe3rvx9/v1
	Dendritic spine analysis	Protocols.io	dx.doi.org/10.17504/protocols.io.e 6nvw1879lmk/v1
	Striatal sections immunofluorescence and analysis	Protocols.io	dx.doi.org/10.17504/protocols.io.n 2bvjnpmxgk5/v1
	Multicolor fluorescence in situ hybridization and analysis	Protocols.io	dx.doi.org/10.17504/protocols.io.3 byl4937rgo5/v1

Drugs and treatment dosing	Protocols.io	dx.doi.org/10.17504/protocols.io.261ger68ol47/v1
Stereotaxic surgeries	Protocols.io	dx.doi.org/10.17504/protocols.io.e6nvw1879lmk/v1
Acute Slice Preparation	Protocols.io	dx.doi.org/10.17504/protocols.io.n92ldr3n8g5b/v1
Current clamp recordings	Protocols.io	dx.doi.org/10.17504/protocols.io.n92ldr3n8g5b/v1
Western blot analysis	Protocols.io	dx.doi.org/10.17504/protocols.io.q26g7mr98gwz/v1
LC-MS/MS Analysis	Protocols.io	dx.doi.org/10.17504/protocols.io.81wgbr1kylpk/v1

Table 1:
The table highlights the details about all reagents, genetically modified strains, software, and source data of the results presented in the manuscript.
