# Supplementary material for: LRRK2 mediates haloperidol-induced changes in indirect pathway striatal projection neurons
Source: Mol Psychiatry. 2025 Apr 23;30(10):4473–86. doi: 10.1038/s41380-025-03030-z (PMC12436163; doi:10.1038/s41380-025-03030-z)
Supplement: Supplementary file 6 — Supplementary Figure 6 [file 41380_2025_3030_MOESM6_ESM.pdf]

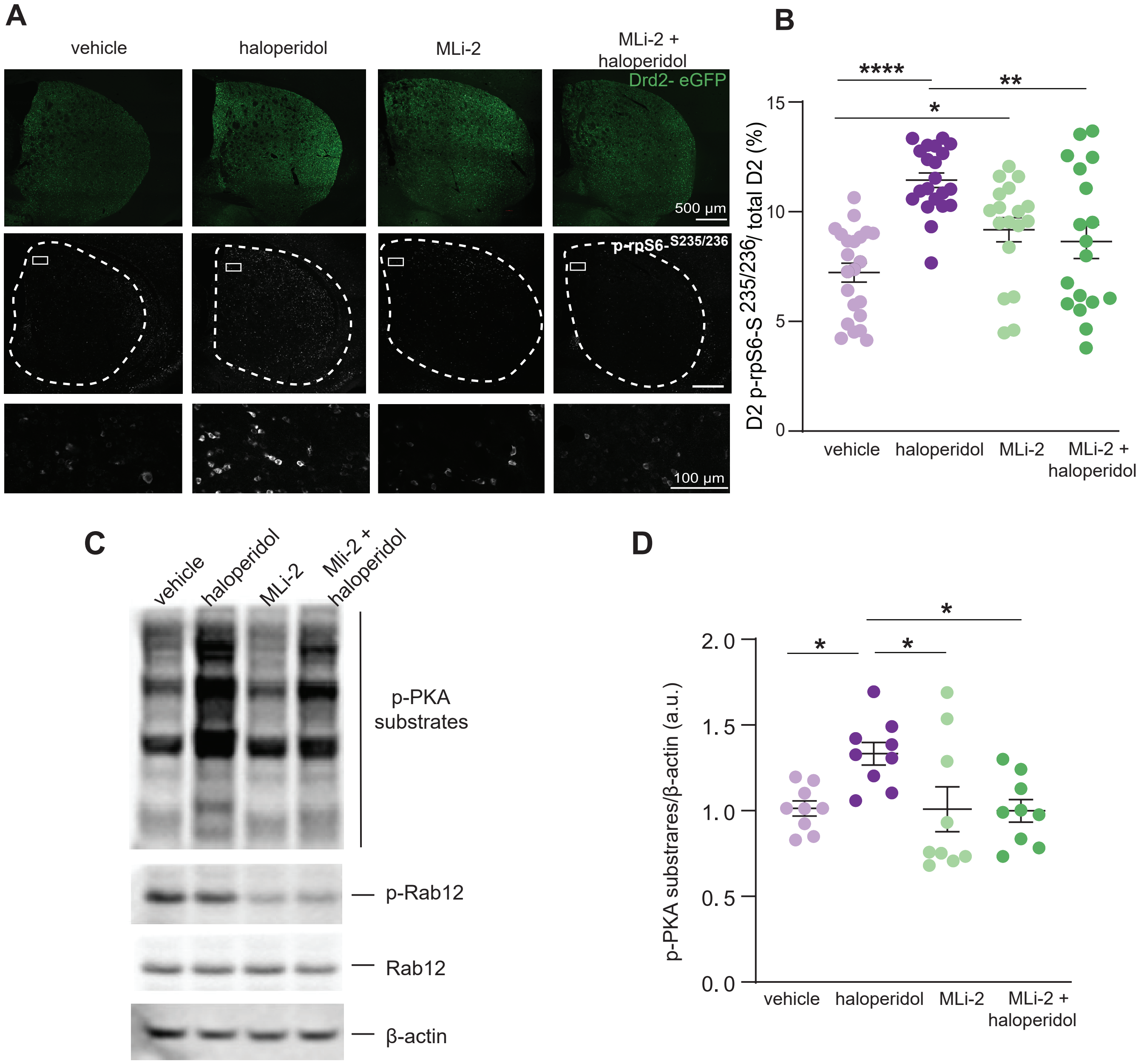

Supplementary Figure 6

Supplementary Figure 6 (linked to Figure 3). LRRK2 kinase inhibition restores haloperidol effects on PKA signaling in iSPNs

A. Representative images showing the distribution of eGFP (top) and p-rpS6 (middle) signal in striatal slices of *Drd2-eGFP* mice treated with vehicle, haloperidol, MLi2, or MLi2+ haloperidol for 7 days. The bottom row shows higher magnification images of p- rpS6 expression. First and second row: scale bar=500 µm; third row: scale bar=100 µm

B. Ratio of p-rpS6 positive Drd2-eGFP cells to total Drd2-eGFP cells. Data reflect mean±SEM. N=18-22 sections/3-4 mice.

C. Western blot analysis in striatal extracts of WT mice treated acutely with vehicle, haloperidol, MLi2, or MLi2+ haloperidol as described in 1A.

D. Quantification of p-PKA signal in C normalized to β-actin. N=9 mice/condition.

Data in B and D reflect mean±SEM. Asterisks show statistical significance for Tukey's multiple comparison tests after one-way ANOVA; *$p < 0.05$. **$p < 0.01$, ****$p < 0.0001$.
